# Crowding Effects across Depth Are Fixation-Centered for Defocused Flankers and Observer-Centered for Defocused Targets

**DOI:** 10.3390/brainsci10090596

**Published:** 2020-08-28

**Authors:** Lisa V. Eberhardt, Anke Huckauf

**Affiliations:** General Psychology, Institute for Psychology and Pedagogics, Faculty of Engineering, Computer Science and Psychology, Ulm University, Albert-Einstein-Allee 47, 89081 Ulm, Germany; anke.huckauf@uni-ulm.de

**Keywords:** real depth, crowding, flanker interference

## Abstract

Depth needs to be considered to understand visual information processing in cluttered environments in the wild. Since differences in depth depend on current gaze position, eye movements were avoided by short presentations in a real depth setup. Thus, allowing only peripheral vision, crowding was tested. That is, the impairment of peripheral target recognition by the presence of nearby flankers was measured. Real depth was presented by a half-transparent mirror that aligned the displays of two orthogonally arranged, distance-adjustable screens. Fixation depth was at a distance of 190 cm, defocused depth planes were presented either near or far, in front of or behind the fixation depth, all within the depth of field. In Experiments 1 and 2, flankers were presented defocused, while the to-be-identified targets were on the fixation depth plane. In Experiments 3–5, targets were presented defocused, while the flankers were kept on the fixation depth plane. Results for defocused flankers indicate increased crowding effects with increased flanker distance from the target at focus (near to far). However, for defocused targets, crowding for targets in front of the focus as compared to behind was increased. Thus, defocused targets produce decreased crowding with increased target distance from the observer. To conclude, the effects of flankers in depth seem to be centered around fixation, while effects of target depth seem to be observer-centered.

## 1. Introduction

One central question in vision science is how we are able to perceive and select distinct objects in a usually cluttered environment full of visual information. The visual search paradigm led to a wealth of insights how scanning the visual scenery for relevant information proceeds. A typical visual search task involves finding a target stimulus among distractor stimuli. In such cluttered displays, extrafoveal vision is usually involved, either to find the target, or to plan a next saccade. Presuming that target and distractors are individuated on a level of early visual representation, it is assumed that the deployment of attention needed to select relevant information is mainly guided by basic visual features [1].

However, early visual representation is in almost all natural scenes restricted by crowding [2]. Crowding describes the phenomenon of reduced recognition performance for peripheral stimuli that are closely flanked by similar stimuli [3], for reviews see [4,5,6,7,8]. Crowding is one of the most important factors limiting peripheral vision and is assumed to evolve from early stages of visual processing hierarchy (e.g., [9]). Thereby, crowding significantly restricts preattentively available information that guide visual search (e.g., [10]). The crowding effect critically depends on the spatial arrangement of target and flankers: crowding is more pronounced the smaller the target-to-flanker spacing and the larger the target eccentricity. Further, effects of spacing and eccentricity interact: the threshold for the effect of target-to-flanker spacing increases with increasing eccentricity (e.g., [3,11,12]). Therefore, to avoid uncontrollable variations of spacing and eccentricity, in a typical crowding task fixation is fixed and the target, defined by its location in relation to the flankers (e.g., the stimulus in the center of a three-character string) needs to be identified.

Usually, visual search and crowding experiments are conducted on two-dimensional planes. However, to understand information processing in the real world, the third spatial dimension, i.e., depth, needs to be considered. Evidence on the impact of depth on information processing in cluttered visual environments is yet scarce, especially for crowding. Most studies worked with virtual depth, i.e., stereoscopic presentations manipulating binocular disparity in order to induce the impression of depth. Here, we present a set of five experiments examining crowding effects in a real three-dimensional set-up.

### Depth Effects on Performance in Visual Search and Crowding Studies

Reviewing studies investigating how depth affects visual search shows that depth information seem to promote efficient visual search. For example, Nakayama and Silverman [13] showed that depth can be segregated in single planes for search. However, having a closer look shows that visual distractors in unattended depth planes still interfere with performance (e.g., [14,15]). Although interference was shown to decrease with increasing disparity between stimuli in a fixation-centered manner (e.g., [16]), it still seems to occur over a fairly wide range (e.g., [17]). In addition, interference among stimuli across depth is often discussed under the assumption of an observer-centered spatial gradient along which stimulus processing gradually changes (e.g., [16]).

The assumption of an observer-centered spatial gradient of attention receives support from findings of prioritized processing of close stimuli: investigations of processing single stimuli in depth for example, show faster and more forceful reactions towards closer targets [18]. Neurophysiological studies show changes in attention-sensitive visual components of the electrophysiological signal when attending to far compared to close stimuli [19]. Using a visual search paradigm, O’Toole and Walker [20] showed, for example, that search for targets presented closer to the observer than distractors is easier when compared to targets presented farther away than distractors (see also, e.g., [16,21]). However, the direction of observer-centered effects in visual search displays seems to be controversial, since some studies find reverse patterns of results with faster reactions toward far targets (e.g., [22,23]).

However, other studies did not find evidence for observer-centered spatial effects: although Plewan and Rinkenauer [15,24] found that surprising depth cues capture attention in a visual search task, no observer-centered spatial dependencies were found. Additionally, Theeuwes et al. [14] did not find any indication for an observer-centered spatial gradient, suggesting that stimulus processing only follows an observer-centered spatial gradient when the task requires focused attention.

In visual search, depth is regarded as one feature capable of guiding attention (e.g., [25]), suggesting that depth is available early during visual processing. Hence, the question arises as to how depth affects crowding. From the current literature, it is difficult to gain a conclusive picture on the impact of depth on crowding, as few studies have investigated the effect of depth on crowding, with those that have yielded apparently inconsistent evidence [26,27,28,29].

Kooi et al. [26] found less crowding when peripheral targets were stereoscopically presented in front and flankers were presented behind the fixation depth plane, as compared to all stimuli on the fixation depth. However, Felisberti et al. [27] also varying target and flanker disparity in opposing direction could only partially replicate this: in one of three observers, depth information helped to recognize the target; in another observer, depth variation reduced crowding only when targets were presented in front of flankers; for a third observer, depth variation did not alter effects when compared to the two-dimensional presentation. Astle et al. [29] systematically varied flanker disparity across a range of depth (±800 arcsec) while keeping targets at the fixation depth. They found a fixation-centered effect of depth: crowding decreased with increased flanker disparity in depth. In addition, though to a weaker extent, Astle et al. [29] also found observer-centered effects: crowding was lower for flankers behind the target as compared to in front. Sayim et al. [28] investigated the effect of target disparity on foveal crowding. The results indicated less crowding for targets in front or behind flankers at the fixated depth. Summing up, the above reviewed studies hint at reduced crowding when the target stimulus is separated in depth from flankers. Nevertheless, conclusions on the effect seem premature, since the spatial arrangements in these studies differed hugely.

The generalizability of the above reviewed findings to information processing in the real world might be limited, because all of these studies used stereoscopy. That way, only one depth cue is varied, namely binocular disparity. However, in real depth, a variety of further cues contribute to the perception of depth. Especially depth information from focus cues, which is, accommodation and defocus blur, are usually invalid in stereoscopic depth presentations and were shown to contribute to distortions of depth perception (e.g., [30,31]). Thus, conclusions on the interference among adjacent objects in real depth space drawn from the aforementioned investigations are limited.

One previous study that was conducted in real depth on the effect of depth on crowding suggests that there is a fixation-centered effect in depth, analogous to the effect of eccentricity in two-dimensional space (2D): Eberhardt and Huckauf [32] presented strings of target and flankers in various defocused depth planes. Their results show that the larger the strings´ distance in depth from the fixation plane, the stronger the crowding effect. The present study extends this approach by separately varying target and flanker distance in real depth. Thus, our aim is to contribute evidence on differential effects of target and flanker depth on crowding effects across real depth. Thereby, a systematic description of effects regarding observer- and fixation-centered references of space and stimulus type (target vs. flanker) is provided. In Experiments 1 and 2, flanker depth was varied while the target was kept at the fixated depth. In Experiments 3–5, the target depth was varied while flankers were kept at fixation. Anticipating the results, for flanker depth a fixation-centered effect on crowding was observed, while the effect of target depth was observer-centered.

## 2. General Method

This research was conducted according to guidelines of the German Psychological Society (DGPs), the German Research Foundation (DFG), and in line with the declaration of Helsinki; all of the studies were considered exempt from formal ethical review. In all Experiments, prior to testing participants were fully informed about the experiment and that no harm is caused from participation. All of the participants gave their written informed consent for inclusion before they took part in the experiment.

### 2.1. Apparatus

**Figure 1:**
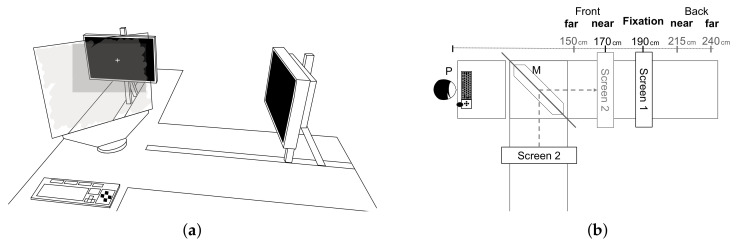
(**a**) Drawing of the experimental setup showing the half-transparent mirror and the two presentation screens in front of the keyboard. (**b**) Scheme of the experimental setup from above. Both screens were adjustable in distance to realize depths as indicated by the scale above. The labels above the scale are the labels used for realized depth planes throughout the paper. During the experiment, one of the two screens (here: Screen 1 which was directly seen through the half-transparent mirror M) was always positioned at the fixation distance of 190 cm. The remaining screen (here: Screen 2 positioned at a distance of 170 cm, which is reflected into the line of sight of the participant P) was positioned at one of the five indicated distances.

The apparatus consisted of two orthogonally arranged screens that were adjustable along a rail [32,33]. The displays of these two screens were superimposed by a half-transparent mirror, which enabled real depth presentation. The experimental setup, as depicted in Figure 1, allowed for stimulus presentation in viewing distances between 150 cm and 240 cm. Both of the presentation screens were 26-inch NEC MultiSync LCD screens (resolution 1440 × 900 px; refresh rate 60 Hz, NEC Display Solutions Europe GmbH, München, Germany). Simultaneous stimulus presentation was controlled by the experimental code, written in MATLAB (Version 7.9, MathWorks, Natick, MA, USA) with the Psychtoolbox extension (Version 3 [34]), running on a Windows XP operating system with a Matrox M9138 LP graphics device.

### 2.2. Stimuli

All of the stimuli were presented on a dark background (black: 0.15 cd/m${}^{2}$). The bright fixation cross (white: 106 cd/m${}^{2}$; size: 0.6° visual angle) was centered on the display. White Landolt rings with four possible opening directions (up, right, down, left) were used as stimuli. Stimulus properties were chosen at the boundary condition of crowding in order to avoid that effects of depth on crowding are obscured by changes in depth perception in the visual periphery (e.g., [35,36]): target stimuli were presented on the horizontal meridian randomly either in the left or right visual field at an eccentricity of 2°. In flanked conditions, one Landolt ring was presented horizontally on either side of the target with a target-to-flanker spacing of 1°. The flankers’ gap position was chosen randomly, given the constraint of incongruency to the target and the other flanker. All of the stimuli had a size of 0.6° of visual angle irrespective of presentation depth.

Defocused stimuli either appeared in a distance near to or far from the fixation depth, each in both directions, in front of and behind the fixation depth of 190 cm. Near distance in front of the fixation depth was at 170 cm from the participant, near distance behind the fixation depth was at 215 cm. This is ±0.06 diopters (dpt), given by the difference between the distances of the fixation and the near depth planes in diopters (e.g., for a distance of 170 cm: $\frac{1}{1.9}-\frac{1}{1.7}=-0.06$ dpt). Far distance was approximately ±0.1 dpt, which was 150 cm in front of the fixation depth and 240 cm behind the fixation depth. (For comparison to studies in stereoscopic depth: The stereo angle ϑ for an inter-pupillary distance of 6.5 cm was for the distances of 150 cm, 170 cm, 215 cm and 240 cm, −1485 arcsec, −742 arcsec, 928 arcsec, and 1856 arcsec, respectively. Stereo angles were calculated by $\vartheta =\frac{p\times \Delta a}{a^{2}}\times \frac{3600\times 180}{\pi }$ [37], where *p* is the inter-pupillary distance, Δ*a* is the depth difference, a is the fixation distance, and $\frac{3600\times 180}{\pi }$ transforms radians to arcseconds.)

Given the experimental setup described above, two screen configurations were realized in the Experiments: either Screen 1 displayed the fixation depth, while Screen 2 displayed the defocused depth (see Figure 1), or vice versa.

### 2.3. Procedure

First, the apparatus and the purpose of the experiment were explained, and the participants were orally informed about their tasks and the duration of the study. After that, the participants were handed out the written information, and they signed the informed consent. Subsequently, participant’s visual acuity and stereovision were screened. Far visual acuity was tested with a Landolt test chart, inclusion criterion was an acuity of minimally 0.7 (decimal scale). Stereovision was tested with red-green random-dot stereograms of the TNO Stereo Vision Test, and with real depth, using the Frisby Stereotest (except Experiment 3). The crowding task was conducted in a dimly lit room (approx. 2.5 lx). Participant’s head position was fixed by a head-chin rest, which was initially calibrated in order to ensure perfect individual central viewing and avoid vertical displacement of stimuli in defocused depth conditions. Prior to the experimental blocks, participants were familiarized with the task and went through extensive practice. Different depth conditions were tested block-wise, with pauses in between. In all experiments, the order of experimental blocks was varied between and balanced across participants.

**Figure 2:**
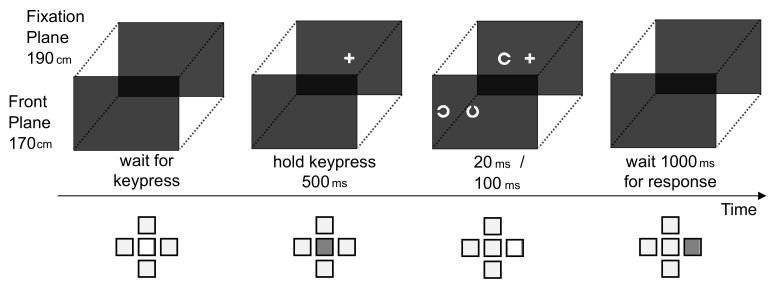
Exemplary sequence of one trial (top) with defocused flankers and response pattern (bottom). A trial was started by pressing the central key. The fixation cross was presented on the fixation plane. After 500 ms of key press, stimuli were presented (for 20 ms in Experiments 1, 2, 4, 5, and for 100 ms in Experiments 2 and 5). Either the target stimulus was presented on the fixation screen, while the two flankers were on the other screen (see example above, Experiments 1 & 2), or vice versa (Experiments 3–5). Aligning the two screens assures that, in the participants sight, stimuli appear in one horizontal line. Participant’s had to indicate the gap position of the target within 1000 ms by pressing one of the four response keys, which were spatially matched to the four possible gap positions.

Participants’ task was to indicate the gap position of the target Landolt ring by keypress. Figure 2 illustrates the sequence of a trial and response behavior. For response, the numerical pad of a usual keyboard was used. The participants started each trial self-paced by pressing a central starting key. The four response keys were arranged cardinally around the starting key (e.g., upper key for upward gap position). After pressing the starting key, the fixation cross appeared. As soon as the starting key was held for 500 ms, stimuli were briefly presented (for 20 ms in Experiments 1, 3, 4, and for 100 ms in Experiments 2 and 5) to avoid the influence of saccadic eye movements (e.g., [38]). Because stimuli were not masked after presentation, the after image was available for stimulus processing. The participants were instructed to release the starting key and press the respective response key as fast and as correct as possible. When reaction time exceeded 1000 ms after stimulus onset, an error sound was played and the response was omitted. The next trial started by pressing the starting key again.

## 3. Experiment 1: Effects of Flanker Direction and Distance in Depth

The aim of Experiment 1 was to investigate the effects of flanker depth on crowding. Because previous studies found fixation- as well as observer-centered effects of flanker depth (e.g., [29]), flanker distance and flanker direction was varied in depth. Flanker distance was varied in two steps, a near and a far depth plane from fixation on which flankers were presented, in order to investigate potential fixation-centered effects. Further, to test for potential observer-centered effects both directions in depth were investigated: flankers were presented in front, i.e., closer to the observer, and behind the fixation depth, i.e., farther away from the observer.

In addition, Experiment 1 aimed at investigating which extent potential effects of real depth are driven by binocular depth information by applying a monocular control condition: If monocularly available defocus blur accounts for differences in depth, the effects of flanker distance and direction in depth should occur similarly under monocular and binocular observation. If differences in depth are mainly driven by binocular depth information, crowding effects across depth for monocular observation should not vary with flanker depth (compare [32]).

### 3.1. Methods

#### 3.1.1. Participants

The sample consisted of 20 participants ($M_{age}$ = 23.45 years, SD = 2.76) with normal or corrected to normal vision, which was screened monocularly and binocularly. Stereovision in the TNO Stereo Vision Test was Med = 60 arcsec (Min = 120 arcsec, Max = 30 arcsed) and Med = 20 arcsec (Min = 40 arcsec, Max = 5 arcsec) in the Fisby Stereotest. The left eye was dominant in six participants.

#### 3.1.2. Stimuli, Design and Procedure

In Experiment 1, flanker depth was varied while the target was always presented at the fixated depth. The performance for flanked targets was tested block-wise, based on flanker depth (far front, near front, fixation, near back, far back) and screen configuration (fixation depth on Screen 1 or Screen 2). Thus, each participant went through ten experimental blocks. The presentation of isolated targets was evenly distributed across the ten blocks. In each block, 48 trials were conducted: four (gap position of the target Landolt ring) ×2 (visual field), ×5 (repetitions) = 40 flanked trials; plus eighr isolated target presentations, given by four target gap positions ×2 visual fields. Moreover, all ten experimental blocks were conducted twice by each participant, once with binocular and once with monocular viewing.

Monocular and binocular data were obtained in two separate sessions, with a pause of at least 15 min. in between. For monocular observation, the non-dominant eye was occluded while the dominant eye was centered. Half of the sample started with binocular, the other half with monocular viewing.

### 3.2. Results and Discussion

Data analysis was performed using IBM SPSS Statistics Version 25 (IBM Corp., Armonk, NY, USA). An alpha level of α = 0.05 was applied in order to report statistical significance in all Experiments.

Crowding effects were analyzed, i.e., the difference between isolated and flanked accuracy in target recognition. To investigate the impact of flanker depth, we conducted a 2×2 repeated measures ANOVA with flanker distance (near vs. far) and flanker direction (front vs. back) as within-subject factors on crowding effects. In addition, one sample *t*-Tests were performed post-hoc in order to compare conditions with defocused flankers to the two-dimensional condition at the fixation depth.

#### 3.2.1. Binocular Observation

**Figure 3:**
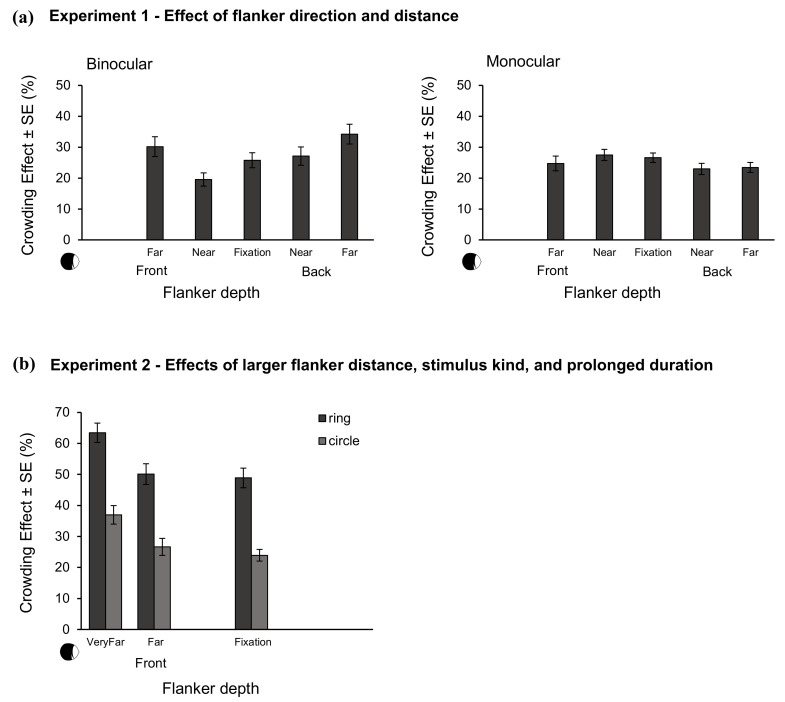
(**a**): Mean crowding effects and standard errors as a function of flanker depth in Experiment 1 for binocular (left) and monocular observation. (**b**): mean crowding effects and standard errors in Experiment 2 as a function of flanker depth and kinds of flankers. The x-axis of the plot is aligned to the plot above to facilitate comparison of result patterns. In both experiments, targets were presented on the fixation depth while flankers were defocused. Observer pictograms on the left side of the x-axis illustrate that data points from left to right are ordered with regard to increased distance from the observer; x-labels refer to the fixation-centered reference labelling used throughout the paper.

In the upper part of Figure 3, crowding effects are plotted as a function of depth for binocular (left) and monocular (right) observation. The accuracy for isolated targets was with *M* = 98.16% (standard error (SE) = 0.49) substantially higher than for flanked targets in all depth conditions. This shows that the presence of the flankers resulted in substantial crowding in all of the tested depth conditions.

There was a significant main effect of flanker distance, *F*(1,19) = 41.54, *p* < 0.01, $\eta_{p}^{2}$ = 0.69, indicating more crowding in far as compared to near distance. Additionally, the main effect of flanker direction was significant, *F*(1,19) = 6.03, *p* = 0.02, $\eta_{p}^{2}$ = 0.24, indicating more crowding when the flankers were behind as compared to in front of the fixated depth. The interaction of distance and direction was not significant, *F*(1,19) = 1.43, *p* = 0.25. These effects were replicated in another study with a sample of *n* = 24 participants. The results can be reviewed in the Figure S1, which can be retrieved from Open Science Framework (OSF) via the link provided at the end of this paper.

In addition to the effects of flanker distance and direction in depth, we compared crowding effects for defocused flanker conditions to the control condition at the fixation depth by Bonferroni-corrected one sample T-Tests. Relative to fixation depth, crowding was significantly lower when flankers were in near distance in front of the fixation depth ΔM = 6.23%, SE = 1.42, *p* < 0.01. Flankers in far distance behind the fixation depth produced significantly more crowding when compared to the fixation depth, ΔM = −8.45%, SE = 2.25, *p* = 0.01. The two other comparisons were not significant (Far front vs. Fixation ΔM = −4.4%, SE = 3.05, *p* > 0.99; Near back vs. Fixation ΔM = 1.34%, SE = 2.08, *p* > 0.99).

Taken together, binocular data revealed a fixation-centered effect, i.e., increased crowding with increased flanker distance, as well as an observer-centered effect, i.e., less crowding for flankers in front as compared behind the fixation plane, of flanker depth on crowding effects.

#### 3.2.2. Monocular Observation

Crowding effects with monocular viewing are given in the top right panel of Figure 3 as a function of flanker depth. Accuracy for isolated targets was with *M* = 94.4 (SE = 1.71) substantially higher than for flanked targets in all of the depth conditions. This shows that the presence of the flankers resulted in crowding in all tested depth conditions.

A 2×2 repeated measures ANOVA on monocular crowding effects for flanked targets with the factors flanker distance (near vs. far) and flanker direction (front vs. back) as within-subject factors revealed no significant effects, neither for flanker distance, *F*(1,19) = 1.57, *p* = 0.23, for flanker direction, *F*(1,19) = 3.86, *p* = 0.06, or for the interaction of distance and direction, *F*(1,19) = 2.43, *p* = 0.14. Thus, with defocus blur as the only possibly available depth cue, flanker depth did not affect target recognition differentially. This replicates earlier findings [32] and is in line with the fact that all of the depths were within the depth of field [39]. Taken together, monocular data suggest that the observed effects of flanker depth are driven rather by binocular depth information than by defocus blur.

Moreover, the results for monocular observation indicate that possibly impaired flanker processing due to defocus blur not explaining the increased processing effort with increased distance, as observed for binocular observation. Thus, it rather may be that difficulties in analyzing stimuli’s spatial position in the third dimension contribute to reduced recognition of targets with increased flanker distance.

## 4. Experiment 2: Effects of Larger Flanker Distance, Kinds of Flankers, and Prolonged Duration

The fixation-centered effect of flanker distance turned out to strongly affect crowding in depth, as was shown in Experiment 1. Therefore, in Experiment 2, we enlarged the spatial distances to follow up this effect and investigate whether increased crowding with increased flanker distance in depth continues with greater distance. With our set-up, this was realized for flanker presentations in front of the target only.

In addition, the results of Experiment 1 suggest that binocular disparity is the critical flanker feature for the effects of depth on crowding. Thus, the question emerges how deeply flankers in depth are processed. Varying the shape of flankers could help to understand how flanker depth interferes with target recognition. Therefore, in Experiment 2 an experimental condition was implemented, in which flanker shape was varied. Precisely, we removed the confusable feature of flankers, which is, the gap in the Landolt ring and applied circle shaped flankers instead. Extensive studies on crowding in 2D showed that reduced similarity between target and flankers results in reduced crowding (e.g., [26,40,41]). Hence, closed rings as flankers should produce less crowding than Landolt rings.

Regarding the impact of the depth of different kinds of flankers, it may be assumed that flanker processing is reduced when flankers are defocused. In that sense, the features of the flankers should become increasingly neglectable with increasing defocus. That is, one might expect that the kinds of flankers should matter the closer their distance to the target ring. However, the investigated depth planes maximally reach the edge of the depth of field, implying no loss of resolution with increased distance in depth, as shown for the monocular condition in Experiment 1. Thus, the effect of flanker depth might not be differential for different kinds of flankers.

Furthermore, to rule out that the short presentation duration in Experiment 1 have restricted the evolution of reliable depth percepts, stimulus presentation was prolonged in Experiment 2 to control for potential effects.

### 4.1. Methods

#### 4.1.1. Participants

The sample consisted of 20 participants ($M_{age}$ = 26.45 years, SD = 7.51) with normal or corrected to normal vision. Stereovison in the TNO Stereo Vision Test was Med = 60 arcsec (Min = 480 arcssec, Max = 30 arcsec), and in the Frisby Stereotest Med = 20 arcsec (Min = 40 arcsec, Max = 20 arcsec).

#### 4.1.2. Apparatus, Stimuli, Design and Procedure

Small modifications of participants position allowed viewing distances between 120 cm and 200 cm in Experiment 2. The fixation distance was set at 200 cm. Again, the targets were presented on the fixation depth plane while flankers were presented either on the same plane or in front. Because of the physical constraints of the experimental setup, only the frontal direction in depth was testable that way. In Experiment 2, the size of all stimuli was 0.5° irrespective of flanker depth. Target-to-flanker spacing was 0.7°. In addition to Landolt rings, circularly shaped rings were used as flankers, corresponding to the Landolt rings in size and line thickness. Two defocused flanker depths were implemented: 160 cm resembling with −0.1 dpt the far distance in the previous experiment (subsequently labelled far), and 120 cm which is with −0.33 dpt at the edge of the depth of field [39] (subsequently labeled veryfar). Stimulus presentation duration was set to 100 ms.

Performance for flanked targets was tested block-wise based on screen configuration and flanker depth (far, very far). Thus, each participant went through four experimental blocks. Trials with isolated targets and with flankers on the fixation depth were evenly distributed across all blocks. Thus, in each block 120 trials were conducted: four gap positions of the target Landolt ring, ×2 visual fields, ×2 kinds of flankers (Landolt ring, circle), ×2 flanker depths (at fixation, defocused), ×3 repetitions, plus 24 isolated target trials given by four gaps, ×2 visual fields, ×3 repetitions.

### 4.2. Results and Discussion

Data were analyzed with a 3×2 repeated measures ANOVA with flanker depth (fixation, far, very far) and flanker shape (Landolt ring, circle) as within-subject factors. Again, the accuracy for isolated targets was with *M* = 96.04 (SE = 1.17) substantially higher than for all flanked conditions. Thus, the presence of flankers resulted in substantial crowding in all tested depth conditions.

In the lower part of Figure 3, crowding effects are plotted as a function of flanker depth for both flanker shapes. The results of the ANOVA revealed a significant main effect of flanker shape, *F*(1,19) = 137.91, *p* < 0.01, $\eta_{p}^{2}$ = 0.88. Further, there was a significant main effect of flanker depth, *F*(2,38) = 21.49, *p* < 0.01, $\eta_{p}^{2}$ = 0.53, indicating increased crowding with increased flanker depth. In detail, post-hoc T-Tests showed no difference between fixation and far depth, ΔM = 1.99%, SE = 1.58, *p* = 0.67, but a significant difference between fixation and very far depth, ΔM = 13.8%, SE = 2.67, *p* < 0.01. The interaction between flanker shape and depth was not significant, *F*(2,38) = 0.44, *p* = 0.65.

As the results show, the effect of increased crowding with increased flanker distance in depth (as observed in Experiment 1) also continues with larger flanker distances. This effect was independent of the kinds of flankers in that circle flankers led to less crowding compared to Landolt ring flankers for all depth distances. Thus, flanker kind seems to determine the base strength of crowding while the flanker deviation in depth adds on this effect. Taken together, Experiment 2 replicates the finding of increased crowding with increased flanker depth and generalizes the effect to larger distances in the depth of field and to longer stimulus presentations.

## 5. Post-Hoc Analysis for Experiments 1 and 2

The results from Experiments 1 and 2 revealed that flankers can interfere even more with target recognition when they deviate in depth from the target at the fixated depth. Thus, one interesting question is to what extent flankers are processed when they deviate in depth. The data of monocular observation in Experiment 1 showed that flanker resolution due to defocus blur has no impact within the present range in depth, replicating earlier findings [32] and supporting what would be expected from the literature on depth of field (e.g., [39]).

We analyzed incorrect responses in Experiments 1 and 2 more closely to further explore the question for flanker processing. Incorrect responses can be separated into false responses (i.e., responses corresponding to the non-presented gap position) and flanker confusions (i.e., responses corresponding to one or the other flanker), since both flankers differed from the target by their gap position. Flanker confusions indicate that flanker features can be processed, but spatial selection is impaired (e.g., [42,43]). If stimuli deviating in depth are processed less elaborated, the proportion of flanker confusions should decrease with increased flanker distance, i.e., in a fixation-centered manner. Observer-centered processing of stimuli deviating in depth should result in a directional difference.

The examined data were normalized to the number of all incorrect responses (compare [44]) to avoid biases due to differences in target processing. Thus, given an incorrect response, the chance level for flanker confusion was $\frac{2}{3}$ and chance level for a false response was $\frac{1}{3}$. We examined flanker confusions descriptively only, because our experiments were not designed to primarily gain these results with only four Landolt ring openings, precluding an elaborated inferential analysis. The data for Experiments 1 and 2 is given in Table 1.

The data show that flanker confusions were substantially higher than expected by chance performance in all flanker depth conditions. Thus, flanker processing is in general impaired only little. This is particularly astonishing because the task irrelevant, i.e., the flanking, stimuli varied in depth. Converging evidence from Experiment 1 and Experiment 2 shows no clear indication for systematic effects of flanker distance or direction, though, a final conclusion would require inferential analysis on a more reliable database. Nevertheless, taking together Experiments 1 and 2, the data suggest that flanker features were processed well. It seems unlikely that impaired feature processing for flankers in depth explains differences among the depth conditions.

## 6. Experiment 3: Effect of Target Direction and Distance in Depth

In the previously described Experiments 1 and 2, we investigated how depth deviation of irrelevant, i.e., flanking, stimuli affects crowding. The aim of Experiment 3 was to examine how depth deviation of the relevant, to-be-identified target stimulus affects crowding. Feature processing of defocused stimuli was affected only few in the previous experiments. This suggests that a defocused target stimulus should also not suffer from decreased resolution. Transferring results from Experiments 1 and 2, processing of a target stimulus should be affected similarly by depth variation: applying the fixation-centered effect of depth distance suggests also for the variation of target distance increased crowding with increased target distance. Regarding the observer-centered effect of direction observed in Experiments 1 and 2 as an effect of relative target-to-flanker configuration in depth (e.g., target in front of flankers), more crowding for targets in front when compared to targets in the back should be expected.

### 6.1. Methods

#### 6.1.1. Participants

The sample consisted of 24 participants ($M_{age}$ = 25.79 years, SD = 7.07) with normal or corrected to normal vision. Stereovison in the TNO Stereo Vision Test, was Med = 60 arcsec (Min = 480 arcssec, Max = 30 arcsec).

#### 6.1.2. Stimuli, Design and Procedure

Target Landolt rings were defocused in front of and behind the fixation depth in near and far distances from the fixation depth, while the flankers were always presented on the fixation depth. As in Experiment 1, a control condition was applied with both targets and flankers on the fixation depth. Thus, five depth conditions were tested. In each depth condition, 160 trials were conducted, given by two contexts for target rings (isolated, flanked), two visual fields (left, right), and four target gap positions (up, right, down, left), repeated five times in both screen configurations.

### 6.2. Results and Discussion

**Figure 4:**
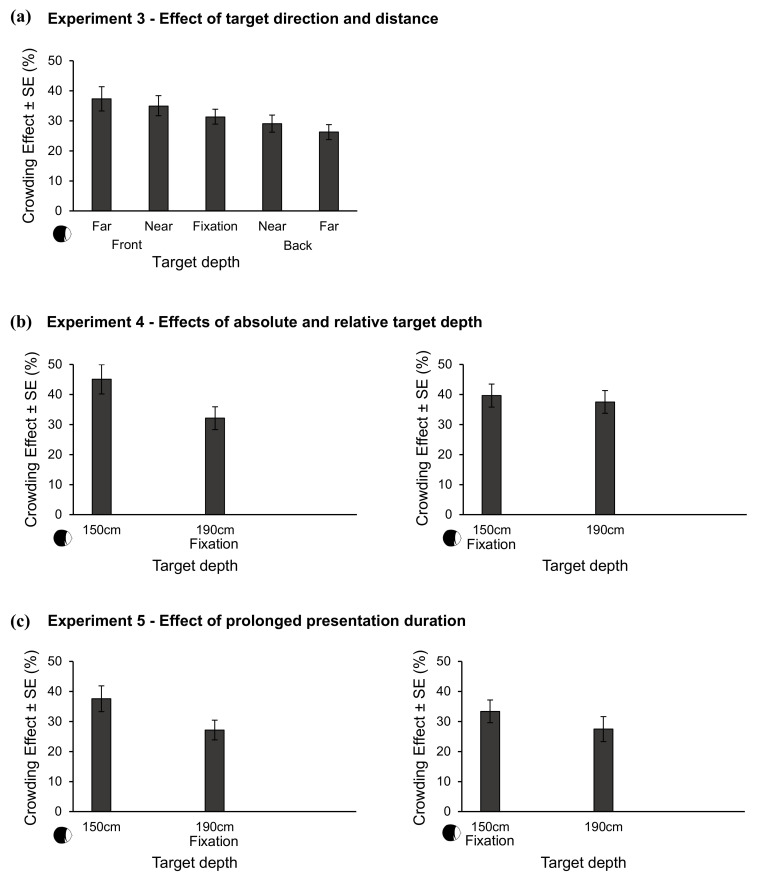
(**a**): mean crowding effects and standard errors as a function of target depth in Experiment 3. (**b**): mean crowding effects and standard errors as a function of target depth in Experiment 4 for fixation at 190 cm (left; as in Experiment 3) and for fixation at 150 cm (right). (**c**): mean crowding effects and standard errors as a function of target depth in Experiment 5 for fixation at 190 cm (left; as in Experiment 3) and for fixation at 150 cm (right). In all Experiments, flankers were on the fixated depth, while target depth was varied. The x-axis of the plots are aligned to facilitate comparison of result patterns. Observer pictograms on the left side of the x-axis illustrate that data points from left to right are ordered with regard to increased distance from the observer; x-labels refer to the fixation-centered reference labeling used throughout the paper.

In the upper part of Figure 4, crowding effects are plotted as a function of target depth. To investigate the impact of target depth, we conducted a 2×2 repeated measures ANOVA with target distance (near vs. far) and target direction (front vs. back) as within-subject factors on crowding effects. The results showed a significant main effect of target direction *F*(1,23) = 12.21, *p* < 0.01, $\eta_{p}^{2}$ = 0.35. The effect of target distance and the interaction between target distance and target direction were not significant, *F*(1,23) < 0.01, *p* = 0.93, and *F*(1,23) = 3.81, *p* = 0.06, respectively.

In addition to the effects of target distance and direction in depth, we compared the crowding effects for defocused target conditions to the control condition at the fixation depth by Bonferroni-corrected one sample T-Tests. The results indicated no significant difference between the condition at the fixation depth and defocused targets at far front, near front, near back, or far back, i.e., ΔM = 6.0%, SE = 3.43, *p* = 0.93, ΔM = 3.55%, SE = 2.31, *p* > 0.99, ΔM = 2.3%, SE = 1.94, *p* > 0.99, and ΔM = 5.02%, SE = 2.09, *p* = 0.25.

Taken together, we observed for defocused targets only an observer-centered effect of target direction, indicating more crowding for targets in the front when compared to in the back. This mimics the results of defocused flankers where there was more crowding when flankers were displayed behind the target. In sum, both of the results could be explained by relative target-to-flanker configuration. Nevertheless, the absence of an effect of target’s distance from fixation is surprising, since, for defocused flankers, distance in depth produced the strongest effects on performance. Therefore, reconsidering the results pattern of Experiment 3, the observer-centered effect of target direction could also be due to the absolute target depth distance from the observer, rather than the relative target-to-flanker configuration in depth. We address this question in Experiments 4 and 5.

## 7. Experiment 4: Effects of Absolute and Relative Target Depth

Rephrasing the pattern of results that were observed in Experiment 3 for target depth, decreased crowding with increased target distance from the observer was found. This raises the question the role of relative depth information like target-to-flanker depth configuration and the role of the relation of stimuli to fixation and the possible contribution of absolute depth to the effects. Therefore, in Experiment 4 the fixation depth was varied in addition to target depth.

### 7.1. Methods

#### 7.1.1. Participants

The sample consisted of 20 participants ($M_{age}$ = 26.3 years, SD = 8.4) with normal or corrected to normal vision. Stereovison in the TNO Stereo Vision Test, was Med = 60 arcsec (Min = 120 arcssec, Max = 60 arcsec) and in the Frisby Stereotest Med = 20 arcsec (Min = 55 arcsec, Max = 5 arcsec).

#### 7.1.2. Apparatus, Stimuli, Design and Procedure

In Experiment 4, only two depth planes were used to keep the overall testing time reasonable: 150 cm and 190 cm. Fixation varied between the two depth planes. Again, target depth was varied: flankers were always presented on the fixation depth plane while targets were presented either on the same or the other depth plane. In addition to angular constant stimulus size of 0.6°, conditions with physical constant stimulus size was tested, for details see Figure S3.

Further, we applied a measure of subjective depth evaluation. Thus, in addition to identifying the gap position of the target, the participants had to indicate the depth plane at which they perceived the to-be-identified target. For this purpose, in the middle between the two depth planes, vertically aligned with and approximately 0.2° below the fixation cross a physical marker was placed as a reference. This was a circle of 0.3°, cut from white paper and mounted on a thin stick. Participants were instructed to indicate subsequent to identifying the target whether the target was presented in front or behind the physical marker by key press with the left hand.

Performance for flanked targets was tested in two blocks based on screen configuration. Trials with isolated targets and with targets on the fixation depth were distributed evenly across all blocks. Thus, in each block 192 trials were conducted: two contexts (isolated, flanked), times2 target depths (fixation, other), plus two target sizes (physical, angular constant), times2 fixation distances, resulting in 12 conditions. In each condition, four gap positions of the target Landolt ring were realized and presented twice in each visual field.

### 7.2. Results and Discussion

In Figure 4, the crowding effects are plotted as a function of target depth. For the sake of consistency data for the control condition with targets of constant physical size are not included in the present paper; the results are presented in the Figure S3 retrievable from OSF via the link provided at the end of this paper.

In order to investigate the impact of target depth, we conducted a 2×2 repeated measures ANOVA with target depth (150 cm, 190 cm) and target-to-flanker configuration (same, different) as within-subject factors on crowding effects. The results revealed a significant effect of target depth, *F*(1,19) = 12.23, *p* < 0.01, $\eta_{p}^{2}$ = 0.39, indicating more crowding for targets at 150 cm as compared to 190 cm. The effect of target-to-flanker configuration and the interaction between target depth and target-to-flanker configuration were not significant, *F*(1,19) = 2.31, *p* = 0.15, and *F*(1,19) < 0.01, *p* > 0.99, respectively. Thus, the results suggest an observer-centered effect: absolute target distance from the observer affects crowding, irrespective of the spatial relation between target and flankers and/or fixation. While the flankers are presented at the fixated depth, the variation of target depth within the depth of field produces systematic changes in crowding, depending on the absolute target distance from the observer: targets closer to the observer result in more crowding.

The results of depth estimation show overall *M* = 50.76% (SE = 0.48) of correct estimation (for details please see Supplementary Materials retrievable from OSF via the link provided at the end of this paper. This does not differ from chance performance of 50%). Thus, depth perception was below a reportable threshold.

## 8. Experiment 5: Effects of Prolonged Presentation Duration

One argument that depth perception was impaired in Experiment 4—as well as perhaps in all experiments reported so far (except Experiment 2)—might be that presentation duration was too short to enable the appearance of depth. Thus, the aim of Experiment 5 was to replicate results from Experiment 4 using a prolonged presentation duration in order to examine whether the observed effects of target depth on crowding are due to restricted presentation duration.

### 8.1. Methods

#### 8.1.1. Participants

The sample consisted of 20 participants ($M_{age}$ = 23.05 years, SD = 2.76) with normal or corrected to normal vision. Stereovison in the TNO Stereo Vision Test, was Med = 90 arcsec (Min = 480 arcssec, Max = 30 arcsec), and in the Frisby Stereotest Med = 20 arcsec (Min = 75 arcsec, Max = 5 arcsec).

#### 8.1.2. Stimuli, Design and Procedure

Stimuli, Design, and Procedure were as described in Experiment 4. However, stimulus duration was prolonged to 100 ms in Experiment 5. Further, the reference marker consisted in Experiment 5 of a small LED lamp on top of the stick in order to increase its visibility in the dimly lit room.

### 8.2. Results and Discussion

In the lower part of Figure 4, the crowding effects are depicted as a function of target depth. Data for the control condition with targets of constant physical size are presented in the Figure S4 retrievable from OSF via the link provided at the end of this paper.

A 2×2 repeated measures ANOVA with target depth (150 cm, 190 cm) and target-to-flanker configuration (same, different) as within-subject factors on crowding effects revealed a significant effect of target depth, *F*(1,19) = 20.47, *p* < 0.01, $\eta_{p}^{2}$ = 0.52, indicating more crowding for targets at 150 cm as compared to 190 cm. The effect of target-to-flanker configuration and the interaction between target and fixation depth were not significant, *F*(1,19) = 1.77, *p* = 0.2, and *F*(1,19) < 0.6, *p* = 0.45, respectively. Thus, the results from Experiment 4 were replicated with a prolonged presentation duration, showing that the presentation duration does not alter the crowding effects in depth.

The results of depth estimation show overall *M* = 49.34% (SE = 0.54) of correct estimation (for details please see Table S1 retrieveable from OSF via the link provided at the end of this paper. Chance level for a two alternative forced choice task is at 50%). Thus, also with a prolonged stimulus duration of 100 ms, stimuli’s depth was below a reportable threshold.

## 9. General Discussion

The aim of the present study was to provide a systematic description of the effect of depth on crowding, regarding stimulus type (target, flankers) and fixation- and observer-centered references in real three-dimensional (3D)-space within the depth of field. Therefore, in Experiments 1 and 2 flanker depth was varied while targets were presented at the fixation depth; in Experiments 3–5 target depth was varied while the flankers were presented at the fixation depth.

### 9.1. Flanker Depth Affects Crowding Mainly in a Fixation-Centered Manner

For defocused flankers, we found both an effect of flanker direction and an effect of flanker distance in depth. Comparing their effect sizes shows that the effect of flanker direction on crowding was clearly pronounced weaker than the effect of flanker distance in depth (see Experiment 1 and Experiment 1.2 reported in the Supplementary Materials). Anyhow, the effect of flanker direction indicated some observer-centered stimulus processing: crowding was increased when flankers were presented behind as compared to in front of the target at the fixated depth. However, flanker depth affected crowding mainly in a fixation-centered manner: with increased flanker distance crowding increased.

The strong effect of flanker distance on crowding is astonishing insofar as flankers are the task irrelevant stimuli within the presentation display. Nevertheless, several observations supported the notion that flankers are not inhibited, but processed to a large extent, irrespective of flanker depth: Flanker shape processing was independent of flanker depth processing, as shown by the effects of kinds of flankers in Experiment 2. Further, this is especially shown by the large proportion of flanker confusions across all flanker depth conditions. Although, flanker features were processed well, crowding increased with increased distance in depth. Increased flanker distance might have increased the processing effort at the cost of target processing, resulting in increased crowding. The fact that, in Experiment 2, a main effect of flanker shape and a main effect of flanker depth, but no interaction, were observed, further leading to an even more specific conclusion: it seems that, with increased flanker distance from fixation, general stimulus processing was not affected (because circle flankers always resulted in less crowding than Landolt ring flankers), but rather, processing of the spatial stimulus configuration and consequential problems to localize the relative positions of the stimuli in depth, might have caused the effect.

Interestingly, a previous study also observed increased crowding when strings containing target and flankers were defocused [32]. Thus, one might speculate whether also in this configuration mainly flanker’s position in depth promoted the effect. Because the effect presents symmetrical around the fixation depth, in a fixation-centered manner, let us assume that it is caused rather by optical than by cognitive processes, as discussed in Eberhardt and Huckauf [32].

At this point, it should not be neglected that both the effect of flanker distance as well as the effect of flanker direction show up in the present study in opposite directions than found by Astle et al. [29]. However, the present study differed from Astle et al. [29] in several aspects, including the method of depth induction (real vs. stereoscopic) and a larger range of defocused flanker depth (approx. ±800–1850 arcsec vs. ±0–800 arcsec). Indeed, the range of flanker depth investigated in the present study exceeds the distance across which crowding across depth was examined so far. Minimal defocused flanker distance (near) in the present set of experiments approximately matches the maximal flanker distance investigated in Astle et al. [29]. Interestingly, some of the descriptive plots of individual participants (i.e., ATA, PVM [29]) in their study also show slight tendencies toward increased crowding for the largest disparities. Hence, one might speculate that increased crowding for far as compared to near flanker distance in depth occurs not only in real but also in stereoscopic depth presentations. This would fit to our data suggesting that increased crowding for far when compared to near flanker distance is a disparity-based effect.

### 9.2. Target Depth Affects Crowding in an Observer-Centered Manner

For defocused targets, we found only an effect of target direction in depth, indicating more crowding for targets in front, i.e., closer to the observer, compared to behind the fixation depth. In Experiments 4 and 5 we further investigated whether this effect of target direction was due to absolute target depth, or target-to-flanker relation in depth. The data suggest a subordinate role of target-to-flanker relation in depth. Rather, the effect of more crowding for targets in front of as compared to behind fixation was driven by absolute target distance from the observer. It seems that flanked targets closer to the observer produce more crowding than flanked targets more distant from the observer, irrespective of flanker depth, and also irrespective of fixation depth (as shown by Experiments 4 and 5). It seems that the spatial reference for the effects of the variation of target depth is the observer’s position in space.

Accordingly, what makes crowding of defocused targets different from crowding by defocused flankers? When varying target depth, a feature of the task relevant stimulus is manipulated while for variation of flanker depth task irrelevant stimuli are manipulated. Theeuwes et al. [14] suggest that observer-centered effects require focused attention. Because the target constitutes the task relevant stimulus this prerequisite might be given.

Another interesting finding in the present data is the direction of the effect of target depth: targets closer to the observer produced more crowding. Similar results were found in some other studies using the visual search paradigm [22,23]. Andersen [22] found increased interference when the target was presented behind distractors. Later, Andersen and Kramer [16] proposed that this could be due to violations of size constancy when adjusting the size of stimuli with different disparities to a constant angular size. This approach means that the size of close stimuli is down scaled, while far stimuli are up scaled to appear of the same size having only different disparities. Our control experiments with stimuli of physical constant size reported in the Supplementary Materials (retrieveable from OSF via the link provided at the end of this paper) showed indeed less crowding when targets were larger compared to flankers and more crowding when targets were smaller. However, further experiments, measuring for example perceived target size, are needed in order to estimate potential effects of violated size expectancy for the results of stimuli of angular constant size reported within this paper.

In addition to this perceptual factor, Maringelli et al. [23] found that attention in a virtual environment is biased toward far stimuli only when no virtual body was present. Because our study was purely perceptual in its nature, requiring only movements of on finger of the participant proprioceptive functions were secondary for the experiment. Although, these factors have possibly contributed to the results that we report, at this point we can only speculate why close targets led to increased crowding.

### 9.3. Crowding and Depth Estimation

In the present study, we investigated whether varying stimulus depth affects crowding. Although, systematic effects of depth on crowding were found, and Experiment 1 confirms that these effects of depth occur after binocular images are combined, Experiments 4 and 5 show that explicit depth estimation was not possible, neither for 20 ms nor for 100 ms of presentation duration. Thus, the present study covers the effects of early depth processing in real three-dimensional space.

These early effects of depth in the present set of Experiments were not sufficient to alleviate crowding substantially in the tested range of depth within the depth of field. Only in Experiment 1 for near flankers in front was crowding reduced as compared to fixation. In most conditions, stimulus selection was impaired by stimulus depth. One possibility is that the spatial configuration of depth cannot be extracted in these early stages of depth estimation. Studies on depth perception have shown that depth of nearby stimuli is averaged when segmentation is not possible (e.g., [45]). However, how does this affect stimulus identification? For near-threshold stimuli it was shown that stereoacuity is degraded by flanking lines (e.g., [46]). The present study indicates that this might also be the case for the identification of supra-threshold stimuli. One might even speculate whether the perception of depth configurations is degraded in crowded stimuli. Anyhow, it seems that crowding can only be alleviated when a segmentation of the stimulus material can be made based on the available depth cues.

The early effects of depth on stimulus selection in real depth described in the present paper might be modulated by other subsequent processes. For example, in visual search, depth can be estimated based on several saccades across depth.

### 9.4. Implications for Visual Search in Real Depth Space

Because saccade planning in visual search relies on peripheral visual information in cluttered environments, crowding is inherent to visual search. Hence, which consequences do the present findings have for visual search in real depth space? Given that crowding can impair estimation of spatial relationships across real depth, visual search in real depth space might take longer because visual selection is impaired. This should result in delayed or inaccurate fixations (e.g., [47]).

The present results seem to be especially relevant for mixed reality settings. Here, situations of crowded information can occur very quickly and depth differences are innate to the presentation devices. The present data showed that when it is not possible to gain information about the spatial relationships in depth, perception is impaired in that crowding increases. One can think of various situations: for example, task that are irrelevant virtual information (operating like flankers) can interfere with identifying real objects, or irrelevant real information can interfere with identifying virtual objects. Thus, the design of augmented reality devices should ensure that the presented information is embedded into the spatial context of the visual scenery to avoid additional interference by spatial uncertainty. 

## Figures and Tables

**Table 1:** Percentage of flanker confusions in all incorrect responses as a function of flanker distance and flanker direction in depth.

		Front			Fixation	Back	
		Very Far	Far	Near		Near	Far
**Confusions M (SE)**							
Exp. 1	Binocular		90.90 (1.94)	90.41 (3.04)	91.82 (1.43)	90.61 (2.74)	85.93 (4.80)
	Monocular		89.55 (1.54)	90.88 (1.37)	87.80 (1.33)	86.79 (2.67)	85.27 (1.53)
Exp. 2		90.27 (1.06)	87.20 (1.74)		89.65 (1.55)		

## Supplementary Materials

The following are available online at Open Science framework following the link https://osf.io/ua6vc/?view_only=76bc0857712445eca199aa91c9f5bf22: Figure S1—Replication of Experiment 1, Figure S2—Experiment 1.2b varying flanker size along with depth, Figure S3—Experiment 4b, Figure S4—Experiment 5b, Table S1—Results of depth estimation task.
